# Theoretical Perspectives in Organocatalysis

**DOI:** 10.1002/chem.202201570

**Published:** 2022-08-16

**Authors:** Nika Melnyk, Iñigo Iribarren, Eric Mates‐Torres, Cristina Trujillo

**Affiliations:** ^1^ School of Chemistry Trinity College Dublin College Green Dublin 2 Ireland

**Keywords:** asymmetric organocatalysis, computational design, computationally-led catalyst design, organocatalysts, prediction

## Abstract

It is clear that the field of organocatalysis is continuously expanding during the last decades. With increasing computational capacity and new techniques, computational methods have provided a more economic approach to explore different chemical systems. This review offers a broad yet concise overview of current state‐of‐the‐art studies that have employed novel strategies for catalyst design. The evolution of the all different theoretical approaches most commonly used within organocatalysis is discussed, from the traditional approach, manual‐driven, to the most recent one, machine‐driven.

## Graphical abstract

**Computational approaches to organocatalysis**: This review is focused on the different computational approaches that have been applied within the organocatalysis field. A huge effort from the theoretical chemistry field has been made in order to prove that *de novo* design of effective catalysts for different types of reactions is a long‐term target, yet a palpable phenomenon.

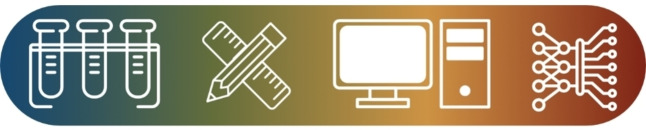

## Introduction

**Scheme 1:**
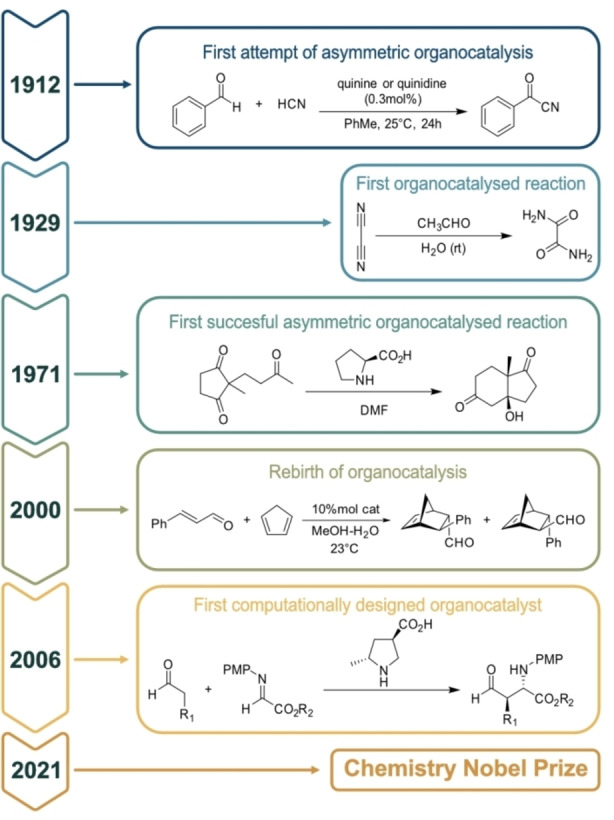
Evolution and historical highlights of organocatalysis.

Catalysis has been a paramount research topic in Chemistry since being separated from ‘alchemy’, proving its value by accommodating many important procedures in this age. Traditionally, it has been largely accepted that transition metal organometallic compounds and enzymes have been the two main types of effective catalysts, in particular in the field of asymmetric catalysts. Consequently, small organic molecules have been rarely used by synthetic chemists as catalysts during the past century, even though the very first pure organocatalyst was reported in 1912. Bredig described a modestly enantioselective alkaloid‐catalysed cyanohydrin synthesis (Scheme 1). Right after, in 1929,^[1]^ Wolfgang Langenbeck reported the formation of oxamides using cyanogen in the presence of aldehyde (Scheme 1). But it was in the 1970s, when Hajos and Perish lead the way to asymmetric organocatalysis, publishing the first and highly enantioselective catalytic aldol reactions using a simple amino acid, proline, as the catalyst.^[2]^

**Figure 1:**
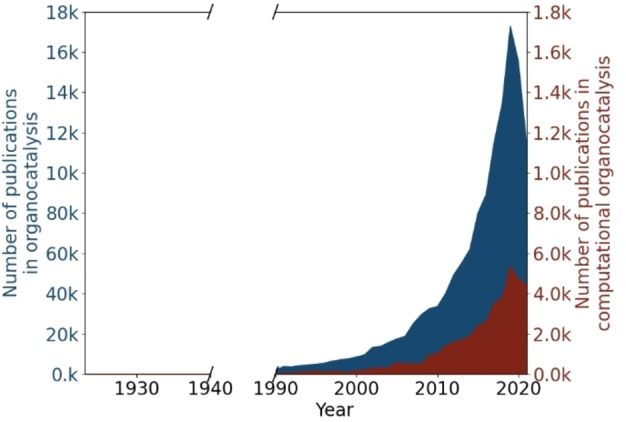
Evolution of the number of papers over time regarding organocatalysis (blue) and computational organocatalysis (red).

Regrettably, despite the significant impact of this compelling milestone in the area of asymmetric organocatalysis, this field remained dormant, for almost three decades. It was during the 2000’s^[3]^ when a change in perception occurred by confirming that relatively simple organic molecules can be highly effective and remarkably enantioselective catalysts of a variety of fundamentally important transformations. This rediscovery induced an immense growth of research activities in organocatalysis, in particular during the last decade (Figure 1). In light of the revolutionary turning point for the field that this asymmetric transformation inspired, it was awarded the Chemistry Nobel Prize in 2021.

While the field of asymmetric organocatalysis has been continuously expanding the ability to unravel the mechanistic details has failed to keep pace, mainly because experimental screening has been one of the most common methods employed in catalyst design. It was not until 2004, when the first asymmetric organocatalytic reaction, the Hajos‐Parris reaction, was studied computationally by Houk and coworkers, elucidating the origin of the activity and selectivity of prolines, later expanded to other amino acids.^[4]^

Subsequently, many reactions have been computationally studied to provide valuable mechanistic insights, leading to different selectivities or effects produced by the catalysts.^[5]^ At that stage, computational chemistry was used as a mere “tool” employed for rationalisation of chemical phenomena. This traditional approach greatly underestimates the predicting potential of computational chemistry.

In 2006, Barbas et al. presented a computational study of a proline‐catalysed Mannich reaction (Scheme 1) successfully leading to the first computationally predicted organocatalyst selective for the *anti*‐configuration. Nowadays and due to the immense development of new methodologies and the evolution of hardware capabilities over the years, the paradigm for the study of organocatalysis and the state of the art is changing towards in silico design rather than experimental screening, showing the promising capabilities of computational chemistry within in organocatalysis.

This review offers a broad yet concise overview of current state‐of‐the‐art studies that have employed novel strategies for catalyst design; a deeper discussion on how these strategies are implemented can be found in a recent focus article from our group.^[6]^

## Discussion

### Manual‐driven approach

**Figure 2:**
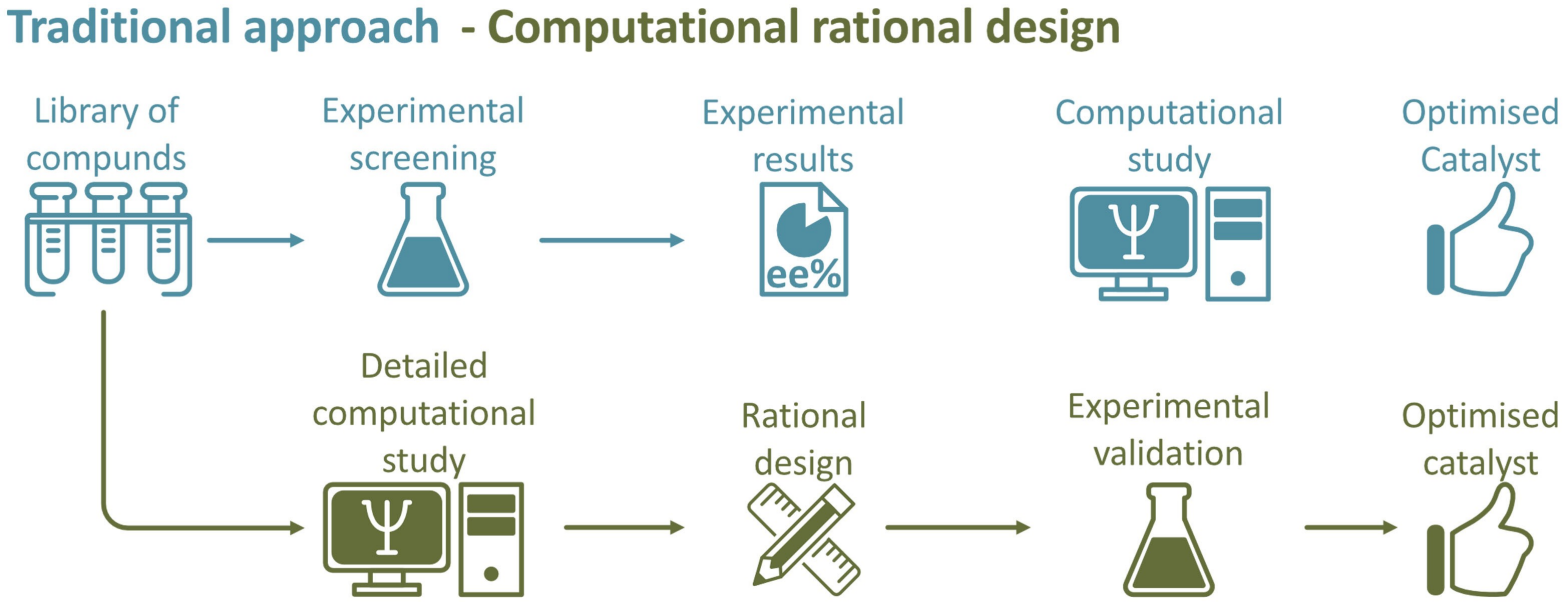
General workflow for the traditional approach (top) and computational rational design (bottom) in catalyst optimisation.

As it has been pointed out, the investigation of organocatalytic systems has been traditionally approached by integrating both theoretical and experimental studies in a collaborative manner (Figure 2, top). This stage is defined by being completely manual, meaning that every step and decision is done by a person, making it very slow and, frequently, tedious. Before, when computational chemistry was emerging, computational studies were performed at the end, as tools to complement, validate or explain experimental findings and mechanistic studies but did not provide any predictive information to aid within catalysts design.[^7^, ^8^, ^9^, ^10^, ^11^, ^12^, ^13^, ^14^, ^15^]

**Figure 3:**
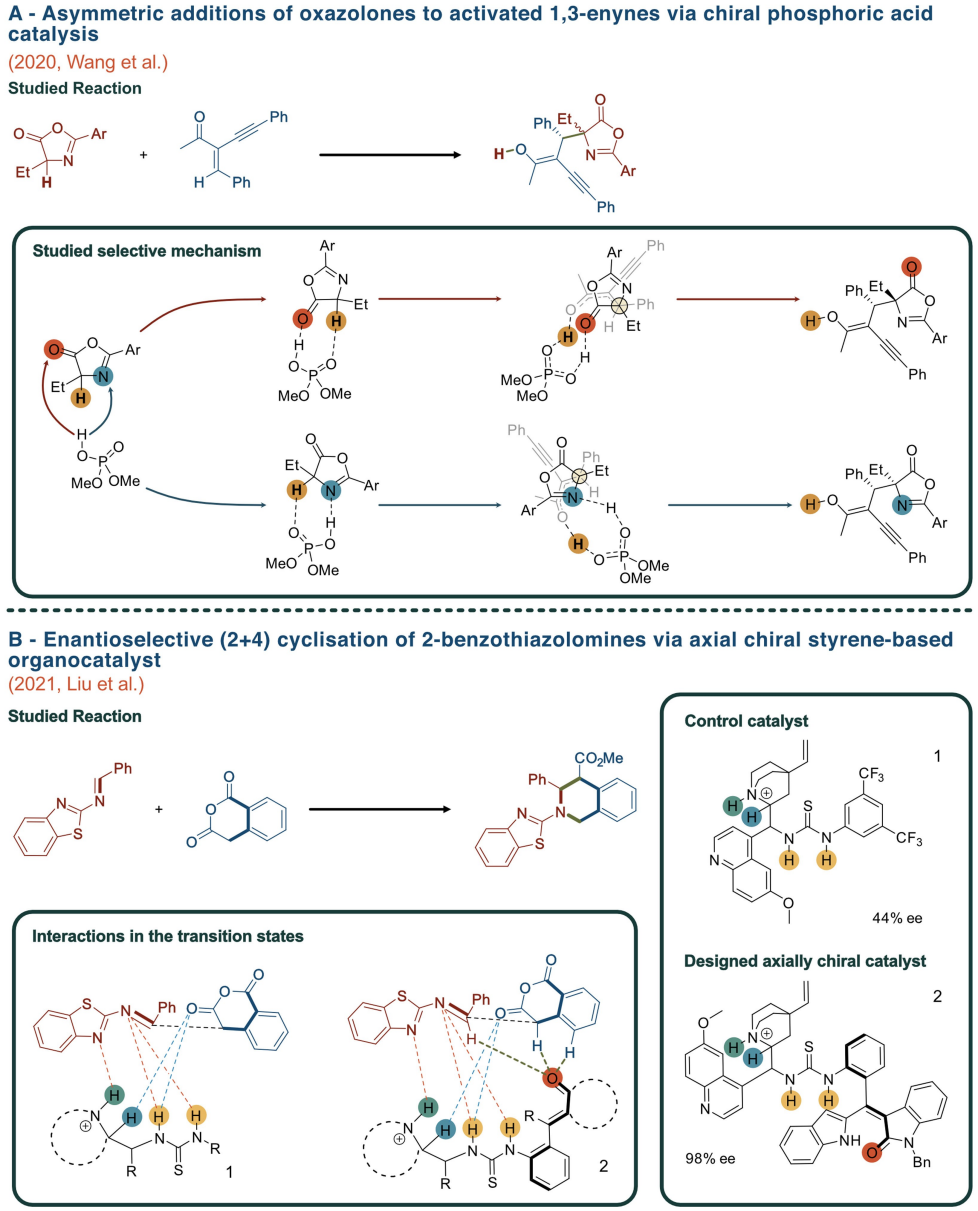
(A) Comparative study of two possible mechanisms of action for the asymmetric addition of oxazolones to activated 1,3‐enynes catalysed by Brønsted acid catalysts: the enol‐type (red pathway) and Münchnone‐type (blue pathway) of mechanism of action. (B) Reaction scheme of the enantioselective cyclisation of benzothiazolomines using a styrene‐based organocatalyst, showing both the control and the rationally designed catalyst as well as the main interactions within the TS using either.

The use of quantum mechanics (QM) to study the possible pathways that may arise from a catalyst of dual nature such as chiral phosphoric acid catalyst, Figure 3(A),^[16]^ provides an explanation of the origin of stereodivergence in the synthesis of trisubstituted allenes by asymmetric additions of oxazolones to activated 1,3‐enynes. Specifically, calculations showed a preference for the more thermodynamically stable Münchnone‐type (Figure 3A, blue pathway) of activation of oxazolones as opposed to the alternative enol‐type (Figure 3A, red pathway).

Traditionally, distereovergence was explained only by sterical effects from the catalyst, however, mechanistic studies revealed that non‐covalent interactions (NCIs) were usually more important at guiding the reaction than mere sterical hindrance.^[17]^

Nowadays, computational chemistry is much more advance and the general workflow has changed over the last five years, (Figure 2, bottom), placing the computational study of the catalyst in first place, afternumerous studies exhibited the ability of computational chemistry to quantitatively calculate the enantiomeric excess (*ee*) of a catalyst; showing how it is governed by the delicate balance between NCIs and the sterical factors present in the different transition states (TSs)involved.[^17^, ^18^]

This *in silico* determination of the intermediates along the current synthetic routes has led to a more profound understanding of the geometrical factors and NCIs that, individually or in a cooperative fashion, favour the production of the compound of interest. Overall, computational studies have revealed that the organocatalysts’ stereoselectivity is mainly determined by the presence (or absence) of electronic, steric and dispersion interactions between the catalyst and the species involved in reactivity (Figure 3).^[19]^

Chiral organocatalysts with superior selectivities can be obtained by modulating these effects, *via* precise selection of the catalyst scaffold and/or fine‐tuning the catalyst ligands. The rational design of chiral organocatalysts by computational means has been integrated to become an essential step towards accelerating the discovery of novel organocatalysts.^[20]^

Since computational chemistry began to predict selectivities of catalysts and lead the rational design process, many computational studies have been performed following this new workflow.[^20d^, ^20e^] In an attempt to overcome the drawbacks of current synthetic routes, Liu et al. recently built upon previous theoretical work to rationally design a styrene‐based chiral organocatalysts for the enantioselective cyclization of 2‐benzothiazolimines.^[19b]^ This was achieved through a rational design approach by introducing a thiourea‐tertiary amine with multiple H‐bonding sites onto an oxindole‐based styrene scaffold, which conferred the newly designed catalyst with enantioselectivities of up to 98 % *ee* (Figure 3B). These examples highlight the importance of rationally designing catalysts for enhancing enantioselectivities by computational means.

## Machine‐driven approach

**Figure 4:**
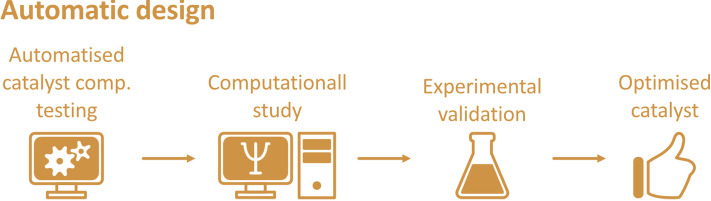
General workflow for automatic approach of catalyst design.

Amongst many different types of strategies for catalyst design, automated design is growing in popularity amongst computational chemists. This approach promises to enhance the efficiency of studies carried out computationally by automating the steps involved; ultimately, this is aimed at accelerating the development process of catalyst design and/or screening (Figure 4).

**Figure 5:**
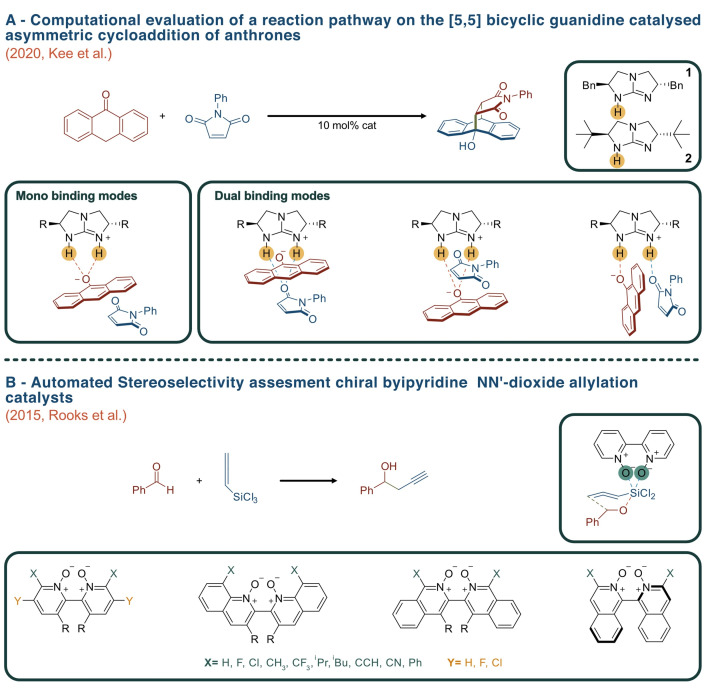
(A) Computational evaluation of the [5,5] bicyclic guanidine catalysed asymmetric cycloaddition reaction of anthrones. (B) automated stereoselectivity assessment of 18 axially chiral bipyridine NN’‐dioxide allylation catalysts.

Chemists began to automate some of the individual steps involved in catalysts design by means of conformational analysis and conformer generation using automated computational programs such as QMTSDock.^[21]^ Developed in 2019 and based on QM calculations the program can study multiple catalysts simultaneously by carrying out an automatic search of the conformational space highlighting any NCI present. Therefore, this approach is particularly valuable for asymmetric catalyst studies where NCIs are often the governing factor that determines the observed enantioselectivity. Additional automated tools exist for the purpose of performing conformational analyses, such as RDKit and CREST (Conformer Rotamer Ensemble Sampling Tool),^[22]^ both being examples of open source cheminformatics tools. QMTSDock, RDKit and CREST have all been used in 2019 by Kee et al.^[23]^ in a computational evaluation of a reaction pathway via generating various conformers for the catalyst and possible TSs as an attempt to address the inability of previous computational DFT studies to account for the enantioselectivity seen experimentally on the [5,5] bicyclic guanidine catalysed asymmetric cycloaddition reaction of anthrones. Kee et al. hypothesised that the reason for such futile outcomes was the inadequate conformation sampling within the study, thus computational tools such as CREST,^[22]^ RDKit and QMTSDock^[21]^ were utilized for the automated conformational analysis compartment of the study, while ORCA^[24]^ was used for geometrical optimisation calculations (Figure 5A).Ultimately, the outcome of this study provided an accurate determination of NCIs involved, concluding that the most favourable pathway is a stepwise conjugate addition‐Aldol sequence via a dual H‐bond binding; in very nice agreement with experimental results. This study highlighted the importance and rewarding nature of integrating automated efforts within theoretical studies that can accelerate not only the accuracy of a calculation but also the geometrical possibilities often overlooked by human error.

As with most computer‐based technologies, the field of automatic design is constantly evolving. Over the years, more studies have embedded these tools into their methodology, often using multiple tools in conjunction. For instance, many of these design software have been integrated with ease into both catalyst design and discovery, allowing theoretical methods to kick‐start the development of new catalytic routes, as opposed to being utilised to complement experimental findings.

Many efforts have been made towards developing a tool that automatically designs and analyses organocatalytic routes. The most illustrative example is the Asymmetric Catalyst Evaluation software (ACE),^[25]^ an open‐source computational tool based on molecular mechanics (MM) methods developed in 2008, used for the virtual screening of organocatalytic asymmetric reactions. In that software energy values of competing diastereomeric transition states are obtained and then evaluated, which are subsequently used to calculate the stereometric excess of anasymmetric reaction. The developers of ACE showcased the reliability of ACE by accurately predicting the enantiomeric and diastereomeric excesses of proline‐catalysed aldolizations. ACE got updated to ACE 2.0 in 2011,^[26]^ which differs from the first release by considering factors such as solvation energies, conformation dependent charge and Boltzmann population. In 2015, this software was successfully implemented by Gerosa et al. for the rational design of chiral pyrrolidine organocatalysts for a Diels Alder cycloaddition. They used the initial hit molecule to search for analogues with improved selectivity. Within days 62 catalysts were screened; allowing ACE to predict the most stereoselective endo and exo catalysts, further confirmed by experiments.^[27]^

With a similar aim, the QM‐based open‐source Automated Reaction Optimiser for New Catalysts (AARON),^[28]^ was developed in 2015. This software automatically constructs and optimises molecular entities, such as intermediates and TSs which govern the efficiency of a catalyst . As of 2020, AARON is part of an open‐source organisation called Quantum Chemistry Automation and Structure Manipulation (QChASM),^[29]^ intended for the automation of complex and large molecular systems. AARON^[30]^ has been integrated into state‐of‐the‐art organocatalytic studies in the last decade: in 2015 Rooks et al.^[30]^ used AARON to carry out an automated stereoselectivity assessment of 18 axially chiral bipyridine NN’‐dioxide allylation catalysts (Figure 5B). Specifically, 18 possible chair‐like catalyst and their respective TSs structures leading to *R* and *S* enantiomer were automatically analysed. Due to the numerous catalysts assessed through this automated methodology, a new origin of stereoselectivity was found and paved the way for the potential computational design of novel catalysts. Similarly in 2016, Doney et al. found new trends in the enantioselectivity of asymmetric propagations by screening a size‐able number of catalysts using AARON; leading to the design of a new catalyst with an *ee* exceeding 99 %.^[31]^

Many other automatized software and packages for catalyst design are being gradually integrated into the field of organocatalysts; specifically the following programmes mentioned have the potential to be applied to the growing field. In 2018, Globally Optimal Catalysts, GOCAT,^[32]^ was developed and described as a package “for global optimisation of catalytic environments for inverse design”. Up to now this package has mainly been used for metal‐based catalysts and enzyme studies. More recently, the Catalyst Virtual Screening (CatVS),^[33]^ was developed in 2018; this is an automated tool that uses Q2MM for virtual screening of substrate and ligand libraries aimed at asymmetric catalysis. CatVS is a highly efficient and user‐friendly tool designed for high‐throughput experiments only requiring mechanistic knowledge to operate, which has found itself very popular amongst the metal catalysis community. In another instance, DEe Novo OPTimisation of In/Organic Molecules (DENOPTIM),^[34]^ an open source Java package which was recently developed in 2019. This software focuses on the de novo design and virtual screening of functional molecules while also specialising in combinational exploration, DENOPTIM is highly diverse in the type of molecules it can work with (inorganic, organic, metastable, and supramolecular). As of now it has been applied in metal‐based catalyst studies^[35]^ such as metathesis;^[36]^ a process which involves the formation of new hydrocarbons via the exchange of carbon‐carbon bonds facilitated by metal catalysts. The most recently developed automatic design software is NaviCatGA,^[37]^ a software intended for catalyst discovery but can also be utilised as a complementary tool for automated programs for mechanistic studies. NaviCatGA is comprised of a genetic algorithm, which is aimed at obtaining an optimal catalyst that meets a set of desired properties. This is achieved by simultaneously optimising activity and selectivity, the main figures of merit which define a promising catalyst. This software has been effectively applied in the field of organocatalysis, where potential catalyst candidates are a diverse combination of different scaffolds and ligands, rendering the search for an optimal catalyst impossible using traditional means. For instance, NaviCatGA has been used for the multi‐objective optimisation of the bipyridine N,N‐dioxide Lewis basic organocatalysts for the asymmetric propargylation of benzaldehyde.^[37]^ It is generally found within catalyst design that the relationship between selectivity and activity is interdependent and disproportionate, where there is often a trade‐off between the two making the design of an ideal catalyst a complex process. NaviCATGA gives the user full freedom in the input of automation, thus the optimisation setup was varied by Laplaza et al.^[37]^ in order to give rise to different structural outcomes. The variation in results allowed for an in‐depth exploration of the steric and electrostatic features which limit and excel factors such as activity and selectivity, leading to the observation of a general trend that aromatic substituents increase the activity of the catalysts, while electron rich substituents play a role in increasing the selectivity. This observation is in line with experimental^[38]^ optimisation of bipyridine N,N‐dioxide Lewis basic organocatalysts which found that aliphatic substituents decreased activity while the ethynyl group improved the selectivity.

**Figure 6:**
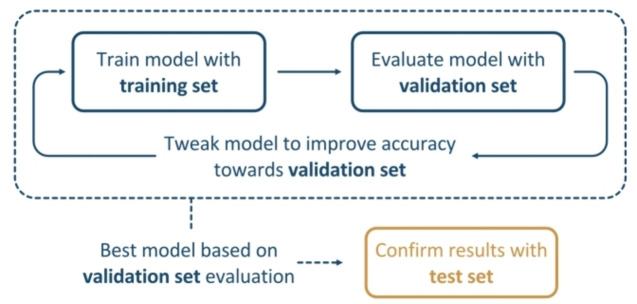
Workflow for the most usual procedure carried out in ML approaches.

Despite the aforementioned advances in the automated design of novel catalysts, the description of the catalytic activity and enantioselectivity in organocatalysis often still requires expensive mechanistic QM analyses or MM approaches. Recently, many efforts have been dedicated to relating molecular properties – or descriptors – such as bond lengths, angles, vibrational modes and charges, with observable reactivity outputs, so as to quantitatively determine activities and selectivities of novel organocatalysts. Identification of patterns within these large datasets for subsequent interpolation becomes an impossible task, and one must rely on machine learning (ML) methods.^[39]^ Most commonly, ML is comprised of a model which is usually trained with a given ensemble of descriptor‐output data ‐ the training set ‐, whose accuracy is subsequently validated with a validation set of catalytic systems with known properties so as to fit to a number of target catalytic systems – the test set (as schematised in Figure 6).

This was applied to the determination of *ee* values by Reid et al., by construction of a training set of MM and DFT‐derived quantitative structure‐reactivity relationships (QSRR) of 367 nucleophile additions to imines catalysed by aromatic phosphoric acids.^[40]^ The relation of the observed activity with the multiple‐term model was determined using linear regression algorithms, which allowed for the identification of the *ee* values of similar catalytic processes within 5 %. A similar methodology was used for the determination of the DFT‐calculated *ee* value of asymmetric propargylation catalysts, whereby the construction of a dataset containing structural parameters from the intermediates preceding and succeeding the enantiodetermining TS resulted in an outstanding R2 value of 0.97 for out‐of‐sample catalysts.^[41]^ Alternatively, neural network methods have been used for the identification of optimal catalytic structures for a set of substituent‐scaffold combinations using a conformer‐derived average steric occupancy descriptor, yielding theoretical *ee* values within 3 % of the experimental.^[42]^ In addition, multivariate linear regression algorithms have recently been used for reaction optimisation, by considering several physical organic empirical parameters, including computationally‐derived NCIs.^[43]^ Hybrid mechanistic/ML methods have also been investigated for the prediction of activation energies with small datasets with high levels of accuracy, as an alternative to pure QSRR methods based on linear regression algorithms.^[44]^

The development of machine learning methods for finding and characterising novel asymmetric organocatalysts is gaining popularity given the myriad of readily available multi‐purpose software, such as PyTorch, Tensorflow or Keras. Furthermore, given the broad range of impact of organocatalysis, most current state‐of‐the‐art organocatalytic strategies opt to rely on variations of known, deeply studied routes. It has been discussed in the literature that novel pathways should not be derived from statistical models based on current models, but use template‐based strategies which allow for a more wide exploration of the field of action without the bias of current literature.^[45]^ In this regard, the increasing implementation of simplified molecular‐input line‐entry system (SMILES) models (which allow for the notation of chemical models using short strings) in automatic design software has had a big influence on the discovery of novel catalysts for a myriad of applications.^[46]^ As more approaches are developed, it is to be expected that ML methods, in cooperation with state‐of‐the‐art automated design software, will play an important role in the accelerated discovery of novel chiral organocatalysts for yet‐to‐explore asymmetric catalytic routes.

## Summary

With increasing computational capacity and new techniques, over the last two decades computational methods have provided a more economic approach to exploring different chemical systems. These developments have brought the synthetic and computational chemists together, and demonstrate the power of this synergy in catalyst design.

In this review, we have summarised all the different computational approaches that have been applied within the organocatalysis community. Starting with the traditional approach in which computational models are used to elucidate the origins of selectivity in organocatalysis. Evolving to the so‐called, computational rational design, in which predictive models are generated involving quantum‐mechanical calculations on complex chemical systems; confirming the power of automatized methodologies in catalyst design; and finally, describing the use of thousands of chemical descriptors to elucidate patterns in multidimensional data.

As it has been clearly stated in this review, a huge effort from the theoretical chemistry field has been made in order to prove that de novo design of effective catalysts for different types of reactions is a long‐term target, yet a palpable phenomenon.

## Conflict of interest

The authors declare no conflict of interest.

1

## Biographical Information

*Nika Melnyk accomplished her Bachelor of Science degree in chemical sciences with medicinal chemistry from the Technological University Dublin (TUD) where she carried out her thesis on the computational analysis of antimalarials containing an endoperoxide bond under the supervision of Dr. Sarah Rawe and Dr. Cristina Trujillo. Her thesis established an interest for computational chemistry and she is currently doing her PhD under the supervision of Dr. Cristina Trujillo focusing on the catalysis of organic reactions through non‐covalent interactions in Trinity College Dublin*.

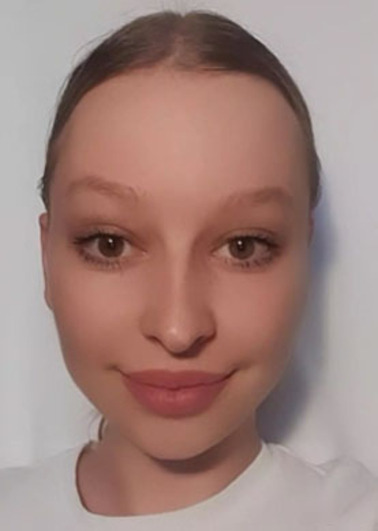

## Biographical Information

*Iñigo Iribarren carried out his chemistry studies at ‘‘Universidad Autónoma de Madrid’’ as well as his master studies in theoretical and computational chemistry. He spent three months at ‘‘Sorbonne Universite’’ in Paris under the supervision of Dr Contreras developing a module for the non‐covalent interaction index software. He carried out his master thesis at ‘‘Instituto de Química Médica (CSIC, Madrid)’’ under the supervision of Prof. Alkorta focused on studying charged dimers linked by hydrogen bonds. Currently, Iñigo is at Trinity College Dublin doing his PhD under the supervision of Dr Cristina Trujillo focused on weak interactions on catalysis*.

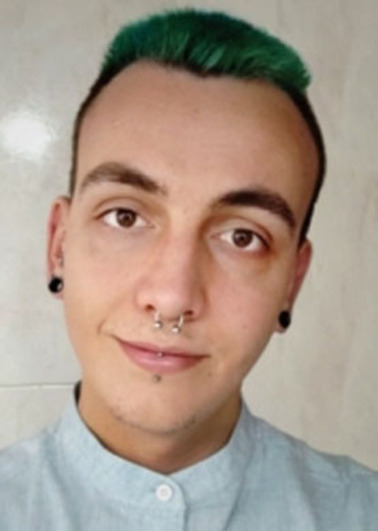

## Biographical Information

*Eric Mates‐Torres performed his undergraduate studies in the “Universitat Autònoma de Barcelona”, specialising in the computational study of organometallic catalysts under the supervision of Prof. Agustí Lledós and Prof. Jean‐Didier Maréchal. After this, he carried out his PhD in the group of Prof. Max García‐Melchor in Trinity College Dublin, focusing on the development of cathode materials for sustainable energy applications. He is currently collaborating with the group led by Dr Cristina Trujillo in Trinity College Dublin*.

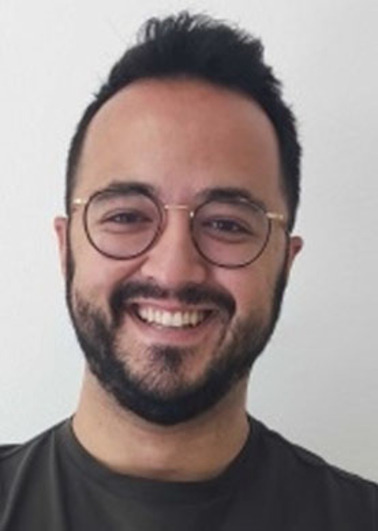

## Biographical Information

*Cristina Trujillo obtained her PhD in Theoretical and Computational Chemistry in 2008 at Universidad Autónoma de Madrid (UAM). During 2008–2016, she held several postdoctoral positions in Spain, Prague and Ireland. She joined Trinity College Dublin in 2019 being awarded with a Science Foundation of Ireland‐Starting Investigator Research Grant. She will join the University of Manchester as a lecturer in Computational Chemistry after summer. She has expertise in fundamental topics within Computational Organic Chemistry. Cristina's research group investigates the application of computational techniques in the design of organocatalysts, as well as the prediction and control of catalytic processes*.

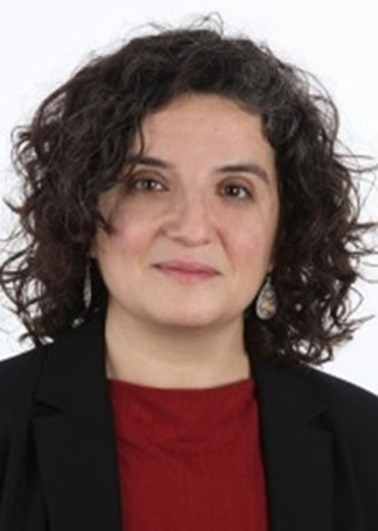

## Data Availability

Data sharing is not applicable to this article as no new data were created or analyzed in this study.
